# Treatment with the C5a receptor antagonist ADC-1004 reduces myocardial infarction in a porcine ischemia-reperfusion model

**DOI:** 10.1186/1471-2261-10-45

**Published:** 2010-09-27

**Authors:** Jesper van der Pals, Sasha Koul, Patrik Andersson, Matthias Götberg, Joey FA Ubachs, Mikael Kanski, Håkan Arheden, Göran K Olivecrona, Bengt Larsson, David Erlinge

**Affiliations:** 1 Department of Cardiology, Skåne University Hospital, Lund, Sweden; 2 Department of Clinical Physiology, Skåne University Hospital, Lund, Sweden; 3 Department of Laboratory Medicine, Skåne University Hospital, Lund, Sweden; 4 Alligator Bioscience AB, Lund, Sweden

## Abstract

**Background:**

Polymorphonuclear neutrophils, stimulated by the activated complement factor C5a, have been implicated in cardiac ischemia/reperfusion injury. ADC-1004 is a competitive C5a receptor antagonist that has been shown to inhibit complement related neutrophil activation. ADC-1004 shields the neutrophils from C5a activation before they enter the reperfused area, which could be a mechanistic advantage compared to previous C5a directed reperfusion therapies. We investigated if treatment with ADC-1004, according to a clinically applicable protocol, would reduce infarct size and microvascular obstruction in a large animal myocardial infarct model.

**Methods:**

In anesthetized pigs (42-53 kg), a percutaneous coronary intervention balloon was inflated in the left anterior descending artery for 40 minutes, followed by 4 hours of reperfusion. Twenty minutes after balloon inflation the pigs were randomized to an intravenous bolus administration of ADC-1004 (175 mg, n = 8) or saline (9 mg/ml, n = 8). Area at risk (AAR) was evaluated by ex vivo SPECT. Infarct size and microvascular obstruction were evaluated by ex vivo MRI. The observers were blinded to the treatment at randomization and analysis.

**Results:**

ADC-1004 treatment reduced infarct size by 21% (ADC-1004: 58.3 ± 3.4 vs control: 74.1 ± 2.9%AAR, p = 0.007). Microvascular obstruction was similar between the groups (ADC-1004: 2.2 ± 1.2 vs control: 5.3 ± 2.5%AAR, p = 0.23). The mean plasma concentration of ADC-1004 was 83 ± 8 nM at sacrifice. There were no significant differences between the groups with respect to heart rate, mean arterial pressure, cardiac output and blood-gas data.

**Conclusions:**

ADC-1004 treatment reduces myocardial ischemia-reperfusion injury and represents a novel treatment strategy of myocardial infarct with potential clinical applicability.

## Background

Reperfusion therapy is the standard treatment of acute myocardial infarction, and restoration of blood flow limits infarct size and reduces mortality. Paradoxically, reperfusion in itself may also cause additional damage to the previously ischemic myocardium, a phenomenon referred to as reperfusion injury [1-3]. The molecular basis for reperfusion injury has not been fully elucidated, but there is evidence for several possible mechanisms of damage including oxidative stress, calcium overload, mitochondrial damage, apoptosis, complement activation and an inflammatory reaction. One aspect of reperfusion injury is the impairment of microvascular coronary blood flow (microvascular obstruction) seen during reperfusion. The development of microvascular obstruction is a multifactorial process attributable to endothelial damage, thrombus formation, neutrophil aggregation, myocyte swelling, capillary spasm and debris from dying cells [4,5]. Microvascular obstruction has been found to be a strong independent predictive marker of postinfarction complications even after adjustment for infarct size [6].

Complement activation is an early event in cardiac ischemia-reperfusion injury [7], and the activated complement system can induce tissue damage both directly and in-directly [8-10]. Directly, the C5b-9 membrane attack complex has cytolytic capacity and has been shown to induce myocardial injury [11,12]. Complement cascade products also appear to injure the endothelium leading to a vicious circle of vasoconstriction, microvascular hypoperfusion and apoptosis [13,14]. Indirectly, the activated complement factor C5a stimulates neutrophils by inducing chemotactic migration [15], aggregation [16,17], and release of cytotoxic products such as proteases, elastases and reactive oxygen species that destroy the cell membrane and cause cell death [16-18]. Neutrophils activated by C5a may also contribute to microvascular obstruction by plugging of the microcirculation [19]. Further support for the importance of neutrophils in ischemia-reperfusion injury is offered by studies demonstrating a cardioprotective effect of either depletion of circulating neutrophils or by inhibition of neutrophil function [17,18]. For these reasons, neutrophils are thought to be important mediators of cardiac ischemia-reperfusion injury [20].

ADC-1004 is a truncated and mutated form of the Chemotaxis inhibitory protein of Staphylococus aureus (CHIPS) [21-23]. It was developed using FIND^®^, a directed in-vitro evolution technology that mimics the natural process of creating protein diversity through recombination [21]. ADC-1004 binds to, but does not activate, the C5a receptor, thereby acting as an effective antagonist [24]. By intervening directly at the C5a receptor, it offers the advantage of exerting its effect on circulating neutrophils, prior to the arrival of the neutrophils at the infarct area. This could be a key to effective anti-neutrophil treatment of myocardial ischemia-reperfusion injury.

We investigated if treatment with ADC1004, according to a clinically applicable protocol, would reduce myocardial injury in a large animal ischemia/reperfusion model. The primary- and secondary end points were reductions of infarct size and microvascular obstruction relative to the area at risk.

## Methods

### Experimental preparation

Healthy domestic male and female juvenile pigs weighing 42-53 kg were fasted overnight with free access to water. The animals were premedicated with Ketaminol (Ketamine, Intervet, Danderyd, Sweden), 100 mg/ml, 0.15 ml/kg, and Rompun (Xylazin, Bayer AG, Leverkusen, Germany), 20 mg/ml, 0.1 ml/kg intramuscularly 30 min before the procedure. After induction of anesthesia with thiopental 12.5 mg/kg (Pentothal, Abbott, Stockholm, Sweden) the animals were orally intubated with cuffed endotracheal tubes. A slow infusion of 1 μl/ml fentanyl (Fentanyl, Pharmalink AB, Stockholm, Sweden) in buffered glucose (25 mg/ml) was started at a rate of 2 ml/min and adjusted if needed. During balanced anaesthesia thiopental (Pentothal, Abbott, Stockholm, Sweden), was titrated towards animal requirements with small bolus doses. Mechanical ventilation was established with a Siemens-Elema 900B ventilator in a volume-controlled mode, adjusted in order to obtain normocapnia (pCO2: 5.0-6.0 kPa). The animals were ventilated with a mixture of nitrous oxide (70%) and oxygen (30%). The pigs were continuously monitored by electrocardiography (ECG). Arterial blood pressure was measured using an MLT0380/D blood pressure transducer (ADInstruments Inc, Colorado Springs, CO, USA). Heparin (200 IU/kg) was given intravenously at the start of the catheterization. A 12 F introducer sheath (Boston Scientific Scimed, Maple Grove, MN, USA) was inserted into the surgically exposed left femoral vein. A 0.021-inch guide wire (Safe-T-J Curved™, Cook Medical Inc, Bloomington, IN, USA) was inserted into the proximal inferior vena cava through the introducer. Using the guide wire, a 10.7 F Celsius Control™ catheter (Innercool Therapies Inc, San Diego, CA, USA) was placed into the inferior vena cava with the tip of the catheter at the level of the diaphragm. Body temperature was measured with a temperature probe (TYCO Healthcare Norden AB, Solna, Sweden) placed in the distal part of the esophagus. The catheter and the temperature probe were connected to the Celsius Control and the system was set to maintain a normal pig body temperature of 38.0°C. A 6 F introducer sheath (Boston Scientific Scimed, Maple Grove, MN, USA) was inserted into the surgically exposed left carotid artery upon which a 6 F FL4 Wiseguide™ (Boston Scientific Scimed, Maple Grove, MN, USA) was inserted into the left main coronary artery. The catheter was used to place a 0.014-inch PT Choice™ guide wire (Boston Scientific Scimed, Maple Grove, MN, USA) into the distal portion of the LAD. A 3.0-3.5 × 15 mm Maverick monorail™ angioplasty balloon (Boston Scientific Scimed, Maple Grove, MN, USA) was then positioned in the mid portion of the LAD, immediately distal to the first diagonal branch. A 9 F introducer sheath (Boston Scientific Scimed, Maple Grove, MN, USA) was inserted into the surgically exposed right jugular vein. A 7.5 F CCOmbo™ continuous cardiac output pulmonary artery catheter (Edwards Lifesciences, Irvine, CA, USA) was inserted into a pulmonary artery. Cardiac output was continuously recorded using a Vigilance™ monitor (Edwards Lifesciences, Irvine, CA, USA). The monitor uses thermal energy emitted by the thermal filament located on the catheter to calculate cardiac output using thermodilution principles. All radiological procedures were performed using an Opescope Pleno™ imaging system (Shimadzu Corp., Kyoto, Japan).

### Experimental protocol

Ischemia was induced by inflation of the angioplasty balloon for 40 minutes. An angiogram was performed after inflation of the balloon and before deflation of the balloon in order to verify total occlusion of the coronary vessel and correct balloon positioning. After deflation of the balloon a subsequent angiogram was performed to verify restoration of blood flow in the previously occluded artery. Twenty minutes before balloon deflation and reperfusion the animals were randomized to treatment with an intravenous bolus dose of ADC-1004 (175 mg, n = 8) or saline (0.9 mg/ml, n = 8). The observers were blinded to the treatment at randomization and analysis. The hearts were explanted four hours after reperfusion and analyzed ex vivo by SPECT and MRI for infarct size, microvascular obstruction and area at risk.

To monitor the plasma concentration of ADC-1004, plasma samples were collected five minutes after administration, at reperfusion, and one, two, three and four hours after reperfusion. Blood levels of leukocytes were measured at baseline and at sacrifice. Plasma levels of Troponin T were analyzed at baseline and at two, three and four hours after reperfusion. Arterial blood-gases were analyzed at baseline, at reperfusion and one hour after reperfusion. Mean arterial blood pressure, heart rate and cardiac output was measured continuously until one hour after reperfusion.

### Dose prediction

Prior to the in-vivo experiment, the potency of ADC-1004 to the C5a receptor was estimated in an in-vitro assay where C5a-induced calcium mobilization was studied by flow cytometry [24]. Pig neutrophils were used and the ability of ADC-1004 to shift C5a-induced concentration-response curves was analysed by Schild-plots, yielding a potency estimate pA2 of 29 nM for ADC-1004 to pig neutrophils. It has been reported that clinically effective concentrations correlates closely with the concentration required for 75% receptor occupancy calculated from the in vitro potency [25], which in the case of ADC-1004, would give an estimated effective concentration of about 90 nM. Thus, the aim was to give a dose that kept the plasma concentration at or above 90 nM throughout the experiment.

### Imaging

Ex vivo imaging of the heart was undertaken according to a previously described protocol [26,27]. The MR and SPECT images were analyzed using freely available software (Segment v1.700, Medviso, Lund, Sweden, http://segment.heiberg.se) [28,29].

### Infarct size and microvascular obstruction assessed by ex vivo MRI

A gadolinium-based contrast agent (Dotarem, *gadoteric acid*, Gothia Medical AB, Billdal, Sweden) was administered intravenously (0.4 mmol/kg) 30 minutes prior to explantation of the heart. The heart was explanted 4 hours after initiation of reperfusion. After explantation, the heart was immediately rinsed in cold saline and the ventricles were filled with balloons containing deuterated water. MRI was performed using a 1.5 T MR scanner (Intera, Philips, Best, the Netherlands). T1-weighted images (repetition time = 20 ms, echo time = 3.2 ms, flip angle = 70° and 2 averages) with an isotropic resolution of 0.5 mm covering the entire heart were then acquired using a quadrature head coil. Approximately 200 short-axis images were generated of each heart, yielding a high resolution for infarct size delineation. The endocardial and epicardial borders of the left ventricular myocardium were manually delineated in short-axis images. The volume of the left ventricular myocardium was calculated as the product of the slice thickness (cm) and the area formed by the delineated borders of the epi- and endocardium. The infarct size was determined as the volume of infarcted myocardium (cm^3^). The infarct volume was calculated as the product of the slice thickness (cm) and the area of hyperenhanced pixels (cm^2^) with a signal intensity above the infarction threshold, defined as >8 SD above the mean intensity of non-affected remote myocardium. Microvascular obstruction was defined as hypointense regions in the core of the infarction which had signal intensity less than the threshold for infarction. The size of the infarct and microvascular obstruction was expressed as percent of the area at risk (AAR), in order to adjust for any differences in AAR.

### Assessment of area at risk by ex vivo SPECT

Single photon emission computed tomography (SPECT) was used to assess the AAR as percent of left ventricular myocardium (LVM). 1000 MBq of ^99 m^Tc-tetrofosmin were administered intravenously 10 minutes before deflation of the angioplasty balloon. Ex vivo imaging was performed with a dual head camera (Skylight, Philips, Best, the Netherlands) at 64 projections (60 s per projection) with a 64 × 64 matrix and a zoom factor of 2.19, yielding a digital resolution of 4.24 × 4.24 × 4.24 mm. Iterative reconstruction using maximum likelihood-expectation maximization (MLEM) was performed with a low-resolution Butterworth filter with a cut-off frequency set to 0.6 of Nyquist and order 5.0. No attenuation or scatter correction was applied. Finally short and long-axis images were reconstructed. The endocardial and epicardial borders of the left ventricle that were manually delineated in the MR images were copied to the co-registered SPECT images. A SPECT defect was defined as a region within the MRI-determined myocardium with counts lower than 55% of the maximum counts in the myocardium [30].

### Analysis of blood samples

Plasma levels of Troponin T were analysed using the Elecsys immunoassay system (Roche Diagnostics Scandinavia, Bromma, Sweden). The white blood cell count (WBC) was measured using a Sysmex XE-5000 automated hematology analyzer (Sysmex Corporation, Kobe, Japan). Blood-gases were analyzed in an automated bench top analyzer (Radiometer Medical ApS, Brønshøj, Denmark). Plasma levels of ADC-1004 were analysed by an ELISA method. In this ELISA, the plates were coated with 2 μg/ml of a mouse mab against ADC-1004. The plates were washed and blocked (3% milkpowder in PBS 0.05% Tween 20) and then incubated with plasma samples. ADC-1004 was detected with 3 μg/ml polyclonal rabbit anti-CHIPS N-terminal IgG (IgG produced by immunization of a rabbit with a KLH-coupled synthetic peptide corresponding to CHIPS N-terminal amino acids 1-14) and horseradish peroxidase (HRP) conjugated goat anti-rabbit IgG (Southern Biotech, Birmingham, AL, USA).

### Ethics

The study conforms to the Guide for the Care and Use of Laboratory Animals, US National Institute of Health (NIH Publication No. 85-23, revised 1996) and was approved by the local animal research ethics committee.

### Calculation and statistics

Calculations and statistics were performed using the GraphPad Prism 5.0 software (GraphPad Software Inc., La Jolla, CA, USA). Values are presented as mean ± SEM. Statistical significance was accepted when p < 0.05 (Mann-Whitney test, two-tailed).

## Results

A total of 20 animals were used. Of those, one was not included due to an anomalous coronary anatomy and one died in ventricular fibrillation prior to randomization. Therefore, 18 animals were randomized between control and ADC-1004. One animal in each group died from pulseless electrical activity. Thus, eight animals in each group were included in the final comparison between ADC-1004 and control. All animals in the treatment group experienced ventricular fibrillation (VF) or pulseless ventricular tachycardia (VT). Six of eight animals in the control group experienced VT/VF. All of these were successfully defibrillated to sinus rhythm and spontaneous circulation. Two animals that received ADC-1004 experienced a transient fall in blood pressure immediately after administration of the drug. This did not occur when the administration was carried out over a period of 1.5-3 minutes. No differences were observed between the ADC-1004 group and the control group with respect to the time from reperfusion to explantation (ADC-1004: 286 ± 2 vs control: 290 ± 4 min, NS).

### Imaging and hemodynamic parameters

**Figure 1:**
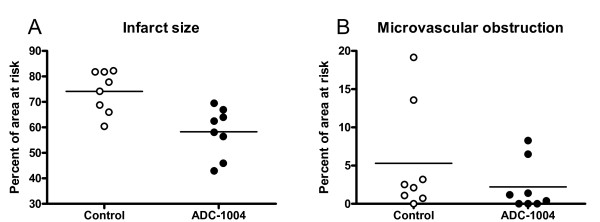
**Imaging**. (**A**) ADC-1004 causes a relative reduction in infarct size of 21%, p = 0.007. (**B**) Microvascular obstruction was similar between the groups, p = 0.23. Horizontal lines denote mean. For microvascular obstruction, the median value was 2.3 percent in the control group and 0.8 percent in the ADC-1004 group.

**Figure 2:**
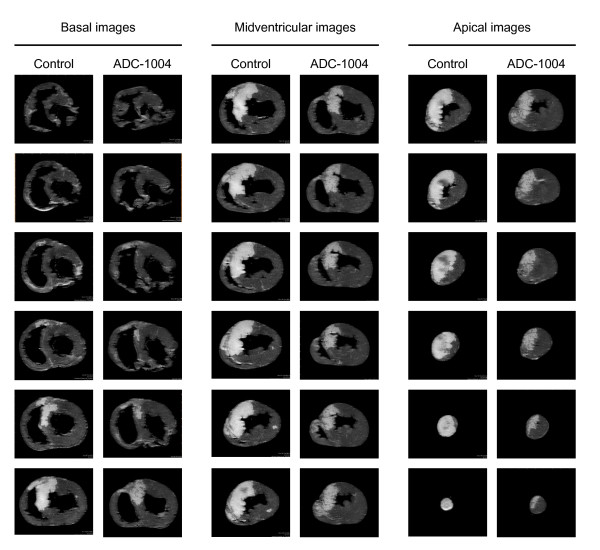
**Infarct size**. Delayed contrast enhanced MR images from one typical animal from each group. Approximately 200 short-axis images, each 0.5 mm thick, are analyzed from every heart. Infarcted myocardium (white) is defined as hyper-enhanced myocardium with signal intensity above eight standard deviations of the signal intensity in the remote myocardium. Microvascular obstruction is defined as hypointense regions in the core of the infarction with signal intensity less than the threshold for infarction.

**Figure 3:**
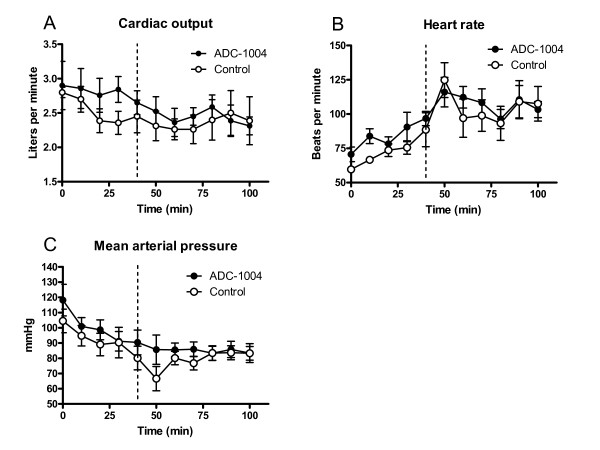
**Hemodynamic parameters**. There were no statistically significant differences in (**A**) cardiac output, (**B**) heart rate or (**C**) mean arterial pressure at baseline, at randomization or onwards. The dashed line marks the onset of reperfusion. Error-bars denote SEM.

ADC-1004 treatment significantly reduced the primary end-point, infarct size relative to the area at risk, by 21% (ADC-1004: 58.3 ± 3.4 vs control: 74.1 ± 2.9%AAR, p = 0.007) (Figure 1A). Infarct size unadjusted for differences in area at risk was also reduced in the ADC-1004 group, although it did not reach statistical significance (ADC-1004: 23.0 ± 3.0 vs control: 27.1 ± 1.4%LVM, p = 0.16) (Figure 2). The area at risk was similar between the groups (ADC-1004: 38.9 ± 3.7 vs control: 37.1 ± 2.7%LVM, NS). The extent of microvascular obstruction was similar between the groups (ADC-1004: 2.2 ± 1.2 vs control: 5.3 ± 2.5%AAR, p = 0.23) (Figure 1B). A large variation in microvascular obstruction was observed in both groups. There were no significant differences in hemodynamic parameters between the groups at baseline, at randomization or onwards (Figure 3).

### Blood sample analysis

Mirroring unadjusted infarct size, the area under the curve (AUC) for Troponin T release was insignificantly reduced by 21% in the ADC-1004 group (ADC-1004: 2823 ± 528 vs control: 3566 ± 615, p = 0.38). The Troponin T detection range was 0.03 - 24 μg/l and in the control group the values were higher than the upper detection limit on a large number of occasions (ADC-1004: 3 vs control: 10 occasions).

**Figure 4:**
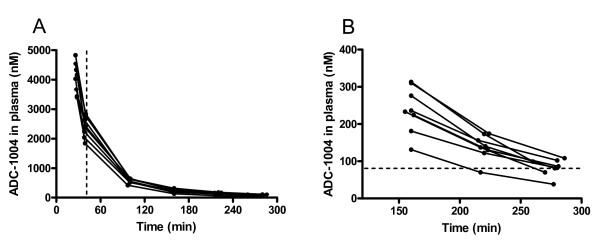
**Plasma concentration of ADC-1004**. (**A**) Plasma concentration over the entire experimental period. ADC-1004 was administered 20 minutes prior to reperfusion, marked by the dashed line. (**B**) Plasma concentration at sacrifice. The dashed line marks the desired level of ADC1004, a concentration that blocks 75% of the C5a receptors on the neutrophils. This level of blockage is considered to be therapeutic.

The mean plasma concentration of ADC-1004 at explantation was 83 ± 8 nM (Figure 4). The degree of reactive leukocytosis in peripheral venous blood were similar between treatment and control at sacrifice (ADC-1004: 149.5 ± 12.1 vs control: 156.5 ± 15.0 percent of baseline value, NS). Blood-gases were similar between the groups (Table 1).

**Table 1:** Arterial blood-gas data

	Control	ADC-1004	P-value
pH - baseline	7.48 (± 0.02)	7.49 (± 0.03)	NS
pH - 40 min	7.36 (± 0.06)	7.41 (±0.03)	NS
pH - 100 min	7.42 (±0.02)	7.43 (±0.03)	NS
Base excess - baseline	4.06 (±1.48)	5.15 (±0.99)	NS
Base excess - 40 min	-1.25 (±3.07)	2.85 (±1.16)	NS
Base excess - 100 min	1.05 (±1.38)	3.25 (±1.10)	NS

Values are expressed as mean and SEM. pH and base excess were similar between the groups throughout the experiment. The unit for base excess is mmol/L and for pH -log_10_{H^+^}

## Discussion

This study evaluated the cardioprotective effect of ADC-1004 treatment, according to a clinically applicable protocol for ST-segment elevation myocardial infarction (STEMI) with administration before reperfusion. Treatment with ADC-1004 was found to significantly reduce infarct size by 21% without any hemodynamic side effects.

The porcine model was chosen because it is a large animal model that resembles the human pathophysiology more closely than rodent models, and also allows for a closed chest model utilising human coronary interventional devices. A percutaneous catheter-based approach allows for induction of ischemia with minimum trauma, operation-induced stress and secondary changes in circulatory physiology. The porcine model also offers a possibility of SPECT and MRI for ischemia and infarct size evaluation. Ex vivo MRI allows for achievement of high resolution images of the myocardial infarction and correlates closely to histology [27,31]. MRI and SPECT are also the gold standard methods of evaluation of ischemia and infarct size in clinical practice. Temperature is known to be a major determinant of infarct size [32]. In order to eliminate spontaneous hypothermia as a confounding factor for infarct size development, a normal core body temperature of pigs (38°C) was maintained. The total ischemia time of 40 min is shorter than in the typical patient with myocardial infarction, which often experience 2-4 hours of ischemia before start of treatment. However, pig infarct progress has been shown to be approximately 7 times more rapid than human [33], suggesting that the current model represents a human STEMI with approximately five hours duration. The faster infarct development seen in pigs is likely due to the lack of collaterals in the porcine coronary circulation.

Treatment with ADC-1004 caused a statistically significant reduction in infarct size of 21%. The extent of microvascular obstruction was similar between the groups, suggesting that the relative contribution of activated neutrophils to the development of microvascular obstruction is less than the contribution of neutrophils to infarct development. The degree of reactive leukocytosis was comparable between the groups, which was to be expected. It is likely that the leukocytes were mobilized during catheterization and early ischemia, prior to administration of ADC-1004. As the mean plasma concentration of ADC-1004 at sacrifice was found to be 83 nM, the aim to keep the plasma concentration at a therapeutic level (i.e. at or above 90 nM) was met for the major part of reperfusion [25]. Thus, as shown in the dose prediction experiment described in the methods section, the circulating neutrophils were blocked at the C5a receptor in the ADC-1004 group and rendered resistant to C5a related activation. The activation has been shown to peak two hours after reperfusion and to rapidly decline thereafter [34]. C5a has also been shown to have a short half-life, with effects resolving within a few minutes [35]. Consequently, a bolus administration of ADC-1004 prior to reperfusion seems to cover the entire therapeutic window for inhibition of C5a-related neutrophil activation. A major advantage with ADC-1004 is that, if it is administered before reperfusion, it will protect the circulating neutrophils before they reach the ischemic zone, thereby avoiding even a brief period of activation. There were no significant differences in hemodynamic parameters between the groups, suggesting that ADC-1004 is safe for administration in patients with acute myocardial infarction.

Several other preclinical studies have evaluated the effect of blocking the C5a component, either by blocking the conversion of C5 to C5a, by neutralising antibodies to C5a or by antagonists of the C5a receptor, over all with findings of cardioprotective effects in animal models [36-39]. Exposure of C5a at a sublytic dose prior to ischemia has also been shown to induce cardioprotection [40], possibly by triggering a preconditioning effect. The preclinical evidence by large, clearly supports a therapeutic possibility in inhibiting C5a related neutrophil activation, which is also in line with the findings in this study.

A few substances have been clinically evaluated recently. The FIRE trial evaluated the effect of inhibition of the neutrophils on infarct size by FX06, a VE-cadherin inhibitor, with the finding of a 58% reduction in necrotic core zone after five days [41]. However, this finding did not remain after four months [41]. The APEX AMI trial evaluated the humanized monoclonal antibody pexelizumab, that binds the C5 component of complement, as an adjunct to PCI in improving 30-day mortality from STEMI [42]. In this trial, mortality was unaffected by pexelizumab treatment. Furthermore, even though pexelizumab had been shown to reduce apoptosis and leukocyte infiltration resulting in reduced myocardial injury in an animal model [39,43], pexelizumab treatment initiated prior to reperfusion failed to favourably affect infarct size in a phase 2 trial [44]. However, an antibody to C5 can neither inhibit the C5a that has already been generated nor can it exert its effect prior to the arrival of the substance in the infarct area. A C5a receptor antagonist, on the other hand, offers the advantage of blocking the receptor on the neutrophils prior to the arrival of the neutrophil in the infarct area.

The C5a receptor is also found on cardiomyocytes, and this receptor activates an intracellular signalling cascade involving protein kinase C isoenzyme delta (PKC-δ) that may contribute to ischemia/reperfusion injury [45]. The substance KAI-9803 is a PKC-δ inhibitor that has been shown to reduce myocardial injury in animal models [46,47], and is now in clinical development [48]. Effects of ADC-1004 on cardiomyocytes could mediate some of the cardioprotective effect by reducing PKC-δ activation, but this needs to be confirmed in future experiments.

## Conclusions

In summary, ADC-1004 treatment, according to a clinically applicable protocol, reduces myocardial ischemia-reperfusion injury. ADC-1004 thus represents a novel treatment strategy of myocardial ischemia-reperfusion injury with potential clinical applicability.

## Competing interests

Dr Bengt Larsson is employed by Alligator Bioscience AB, Lund, Sweden. The other authors declare that they have no competing interests.

## Authors' contributions

The authors have contributed as follows: Conception and design (BL, DE and JVDP), animal experimentation (JVDP, SK, PA, MG and GO), image acquisition (JU, MK and HA), analysis and interpretation (JVDP, BL and DE), drafting of the manuscript (JVDP, BL and DE), critical revision for important intellectual content (all authors), final approval of the manuscript (all authors).

## Pre-publication history

The pre-publication history for this paper can be accessed here:

http://www.biomedcentral.com/1471-2261/10/45/prepub
